# Development and validation of the airway surgery enclosure for high-risk aerosol-generating airway procedures: a bench and clinical study

**DOI:** 10.1038/s41598-025-03705-1

**Published:** 2025-07-02

**Authors:** Neil K. Chadha, Jason Powell, Katharina Leitmeyer, Mark Felton, Alberto Baldelli, Michael Rooney, Fraser G. L. Parlane, Robert Purdy

**Affiliations:** 1 Division of Pediatric Otolaryngology-Head and Neck Surgery, BC Children’s Hospital, University of British Columbia, 4480 Oak Street, Vancouver, BC Canada; 2 Translational and Clinical Research Institute, Faculty of Medical Sciences, Newcastle University, Newcastle Upon Tyne, UK; 3 Division of Pediatric Otolaryngology-Head & Neck Surgery, University Children’s Hospital of Basel, Basel, Switzerland; 4 Faculty of Food and Land Systems, University of British Columbia, Vancouver, Canada; 5 Department of Pediatric Anesthesia, BC Children’s Hospital, University of British Columbia, Vancouver, Canada; 6 Department of Chemistry, The University of British Columbia, Vancouver, BC Canada; 7 Stewart Blusson Quantum Matter Institute, The University of British Columbia, Vancouver, BC Canada

**Keywords:** Airborne viruses, Airway enclosure, Laryngoscopy, Protective equipment, COVID-19, Translational research, Outcomes research

## Abstract

Procedures on the upper airway in patients with respiratory viruses are considered to carry the greatest risk of infection spread to operating room personnel through aerosolization. Appropriate personal protective equipment must be worn, but availability varies worldwide and resources may be limited. We describe the development, validation, and safe implementation of a reusable enclosure with an inexpensive, acrylic design, for use in high-risk airway procedures. Examples of common yet high-risk, aerosol-generating procedures performed with the Airway Surgery Enclosure (ASE) include laryngo-bronchoscopy, suspension laryngoscopy for removal of airway lesions, and rigid bronchoscopy including airway foreign body removal. The ASE demonstrated an 87–94% reduction in aerosolized particle concentration compared to ambient room levels. Bench testing validated the containment capability through laser-based particle imaging and air sampling, while clinical evaluations confirmed ergonomic feasibility and usability. While the ASE provides significant reductions in aerosol exposure, implementation challenges include integration with existing operating room workflows, material durability over repeated sterilization cycles, and cost considerations for widespread adoption. Further studies are needed to assess long-term clinical effectiveness and user adaptability.

## Introduction

The novel SARS-CoV-2 virus causing the infection COVID-19 was declared a worldwide pandemic in March 2020 by the World Health Organization. The airway of infected patients has been shown to carry the highest viral load^1^. Otolaryngology airway procedures, are therefore considered to carry the greatest risk of spreading this infection to healthcare workers^2–4^. For this reason, specific measures should be considered to protect health care workers during surgical procedures on the upper airway from the spread of infections in any future viral outbreaks.

Previous physical barrier designs, such as rigid intubation boxes and negative-pressure containment chambers, often posed challenges related to operator access, maneuverability, and effective aerosol containment. Rigid boxes, for example, restricted hand movement and made precise airway procedures more difficult. Additionally, many earlier designs lacked efficient ventilation systems, leading to particle buildup within the enclosure itself. These limitations underscored the need for a more adaptable and ergonomically friendly solution, which the Airway Surgery Enclosure (ASE) addresses by incorporating flexible access points, improved airflow control, and an optimized negative-pressure design.

We describe the development, validation, and a safe implementation strategy for a novel, inexpensive, reusable, acrylic, custom-designed enclosure for use during high-risk, otolaryngologic airway procedures during the COVID-19 pandemic. These procedures include endo-laryngoscopy, suspension laryngoscopy, and rigid bronchoscopy. This non-commercial and open-source design can be added to the arsenal of techniques described and available to otolaryngologists worldwide. The use of this device, and/or future modified versions, will improve safety during high-risk, aerosol-generating procedures in any future pandemics and can be applied to other aerosol-transmissible diseases beyond COVID-19.

We hypothesize that we can design and validate an airway surgical enclosure that can effectively prevent aerosol-transmissible to healthcare workers during high-risk surgical procedures.

## Methods

### Development of the airway surgery enclosure

Dr. Hsien Yung Lai, an anesthesiologist from Mennonite Christian Hospital in Taiwan, designed the Aerosol Box to protect healthcare providers from risk of COVID-19 from SARS-CoV-2 virus exposure during the intubation process^5^. Various modifications to Lai’s box design have been attempted to improve the ergonomics for the intubation provider, but these boxes provide direct droplet protection only and are not sealed units protecting all operating room personnel from fine aerosolized particles^6,7^. In parallel, the use of disposable plastic sheets as shields has been studied for application in aerosol-generating surgical procedures on the airway mucosa^8,9^. Our group recognized the potential to combining the advantages of a rigid container (as in the Aerosol Box) with the benefits of a sealed enclosure (as in the tent-draping techniques). This concept led to a collaboration between Pediatric Otolaryngology and Anesthesia at BC Children’s Hospital together with the Department of Engineering at the University of British Columbia, to design and build a fully-enclosed, clear, acrylic, reusable ‘operating box’. Clear acrylic panels were selected due to their availability, low material cost, and ease of assembly compared to alternative materials. Both internal and external faces can be sanitized using alcohol-based wipes, such as those containing isopropyl alcohol. The ASE underwent several iterations, models, and simulations to successfully design a structure that consistently provided unrestricted access for the surgeon, surgical assistant, and anesthesiologist across a variety of surgical airway procedures, while reducing the risk of viral aerosol transmission to operating room personnel during aerosol-generating medical procedures (AGMP).

**Fig. 1:**
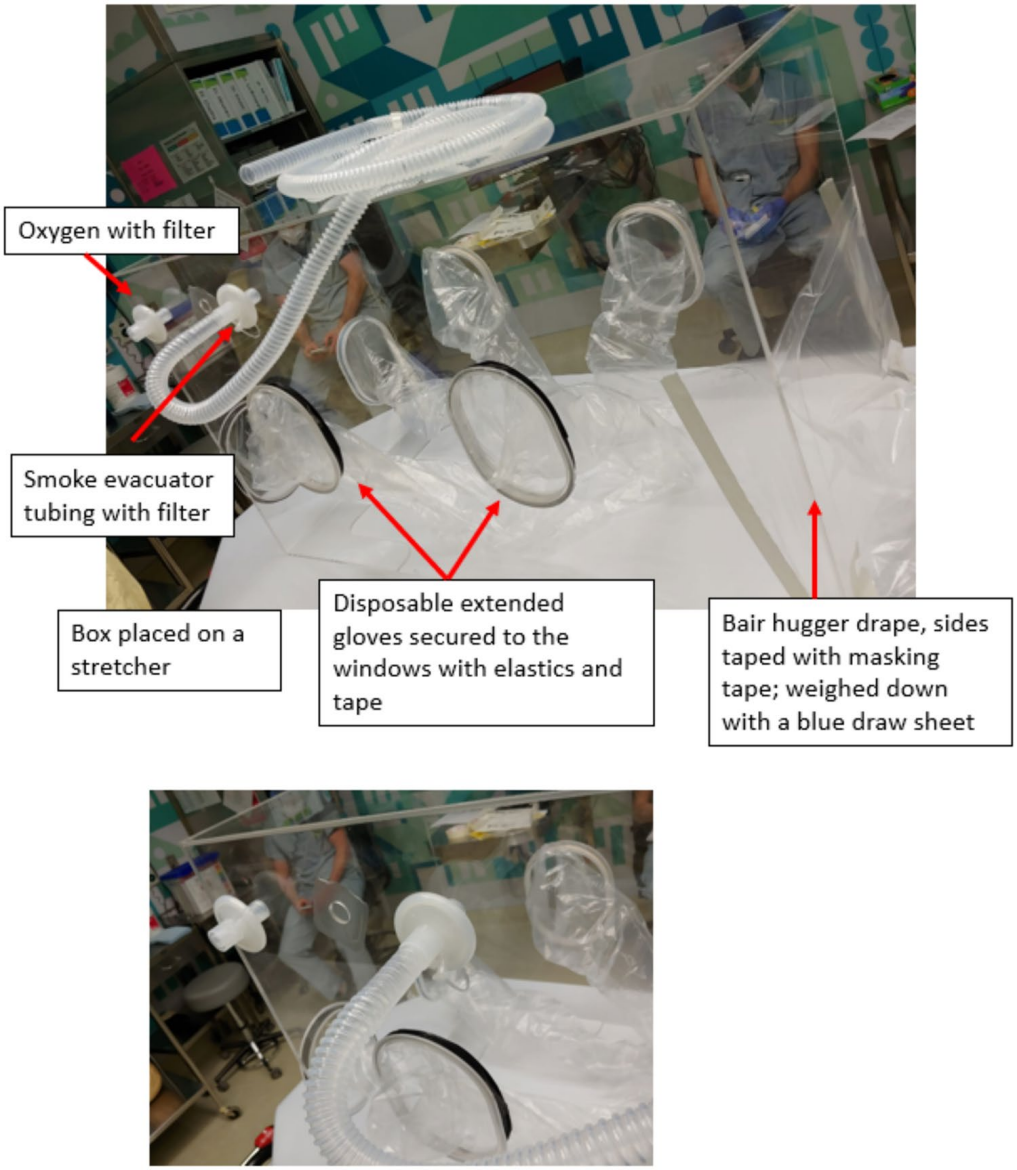
Photo of box labelling arms and how attached.

**Fig. 2:**
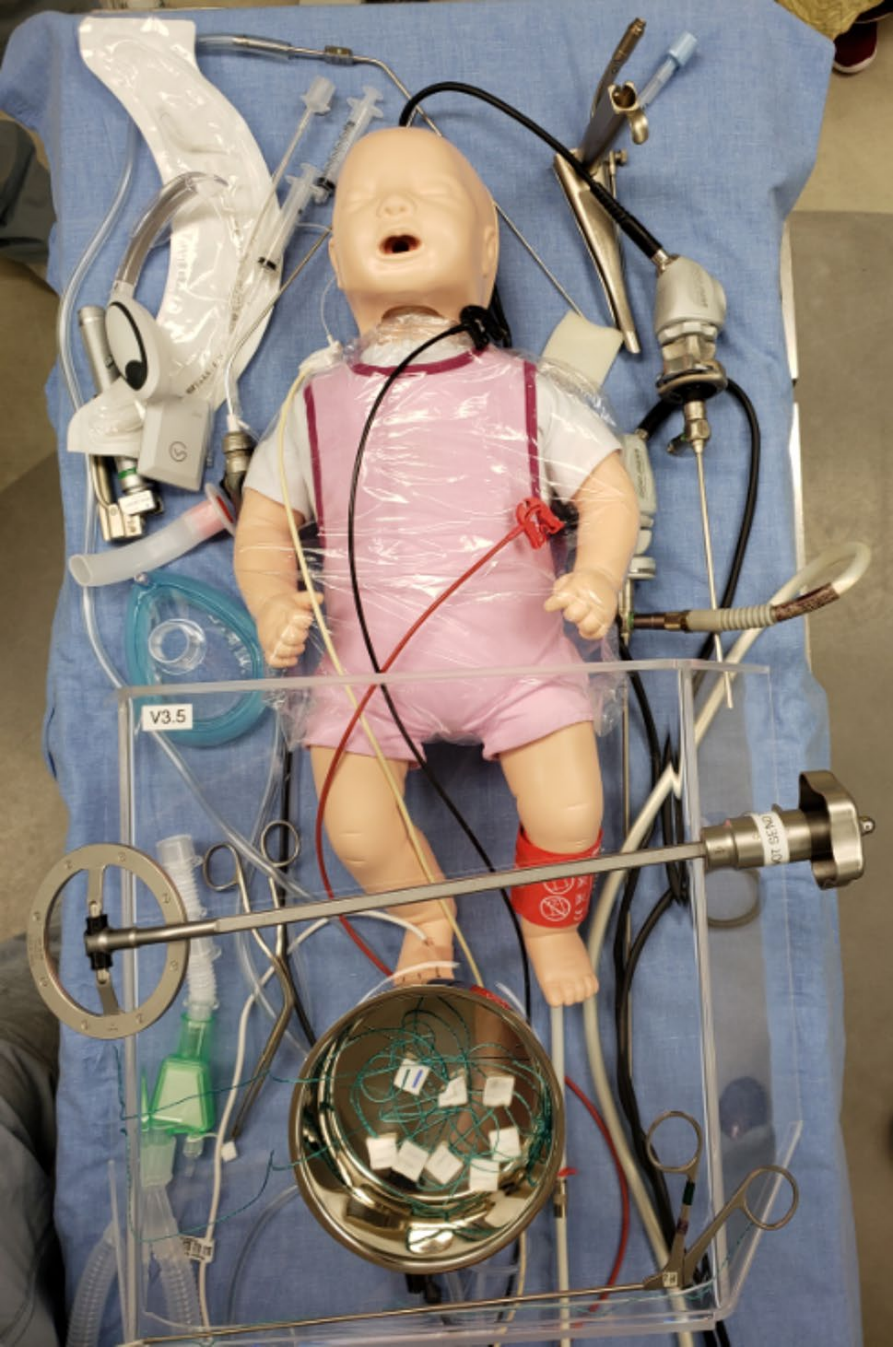
Bed before box placed with manikin and instruments in position.

The ASE was designed by UBC Mechanical Engineer Michael Rooney and manufactured by Walsh Plastics Ltd. in Burnaby, BC, Canada. The panels, corner gussets, and interior table were made from 3/16 in thick Acrylic. The arm hole extrusions and lips were made from 1/4 in and 1/8 in respectively. All pieces were cut by a CNC router table, and seam glued with methylene chloride (acrylic glue). The enclosure integrates plastic arm sheaths that allow the surgeon, assistant, and anesthetist to perform procedures within the enclosure. The cavity of the enclosure is sealed from the rest of the operating room, with the exception of inputs for oxygen delivery and outlets to suction evacuation, thereby generating a negative pressure enclosure (Fig. 1). Oxygen and suction parameters were standardized across tests and the flows were measured frequently. The foot-end of the box is closed over the patient using taped plastic drapes. All required instruments must be positioned in the box before the procedure begins (Fig. 2). The ASE also integrates a removable shelf for instruments and laryngeal suspension, if needed. If additional or unanticipated instruments or equipment are required during the procedure, they are to be introduced under the draping at the foot-end of the bed, which will briefly break the negative pressure field. The herein described version of the box was created and validated for use in pediatric patients with shoulder girth up to 45 cm, which would accommodate most pediatric subjects^10^.

### Testing of aerosolization protection

A comprehensive bench-testing experimental procedure was developed with the aim of measuring, at different points and with different devices, the aerosol generated around the enclosure. The purpose of these tests were to: 1) identify the presence of any leaks in the chamber; 2) measure the concentration number and the mass of airborne particles while performing different activities involving the ASE; 3) identify and explore the benefits of using the ASE while performing some tasks typical airway maneuvers.

**Fig. 3:**
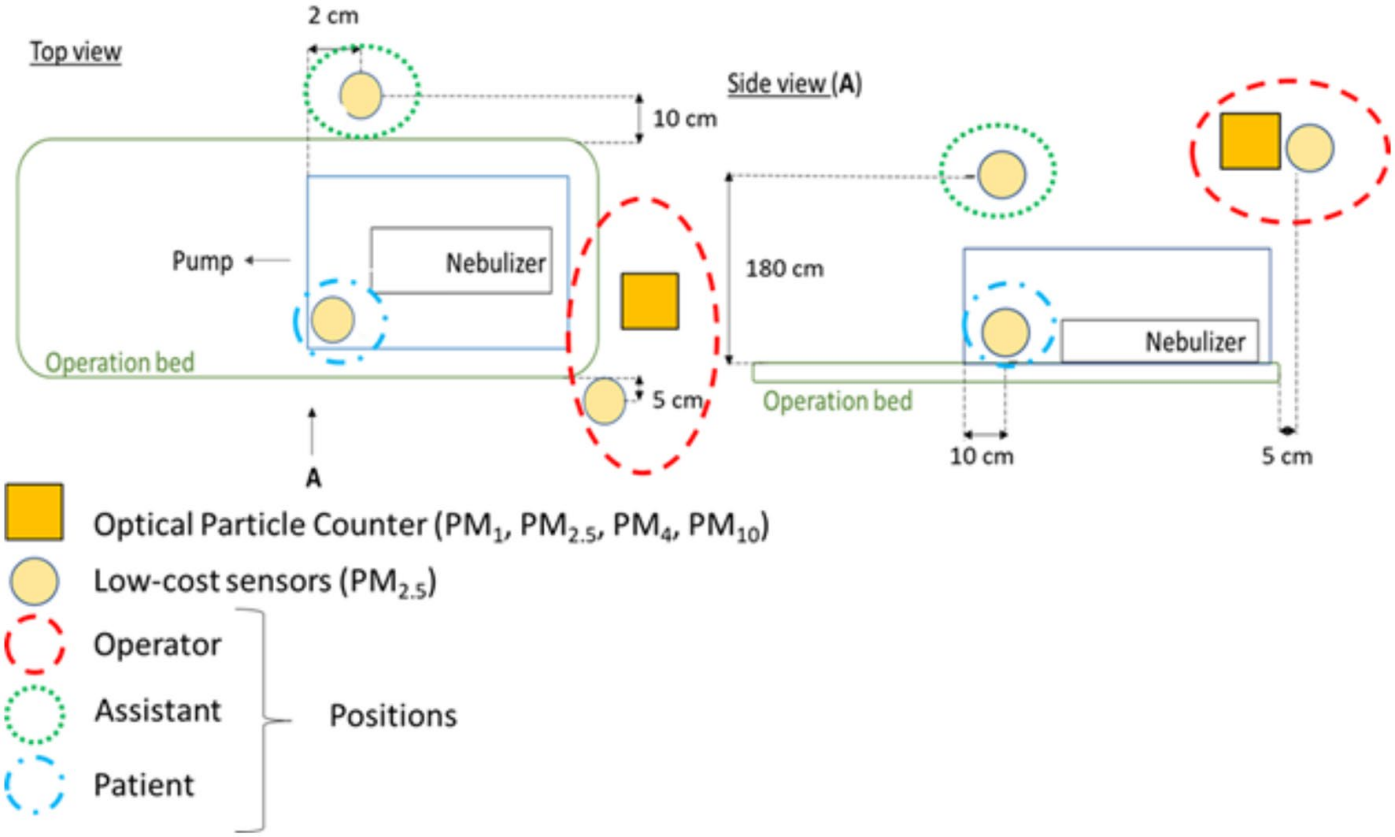
Schema of the locations of the low-cost sensors, in yellow, relative to the nebulizer, enclosure, and bed.

**Fig. 4:**
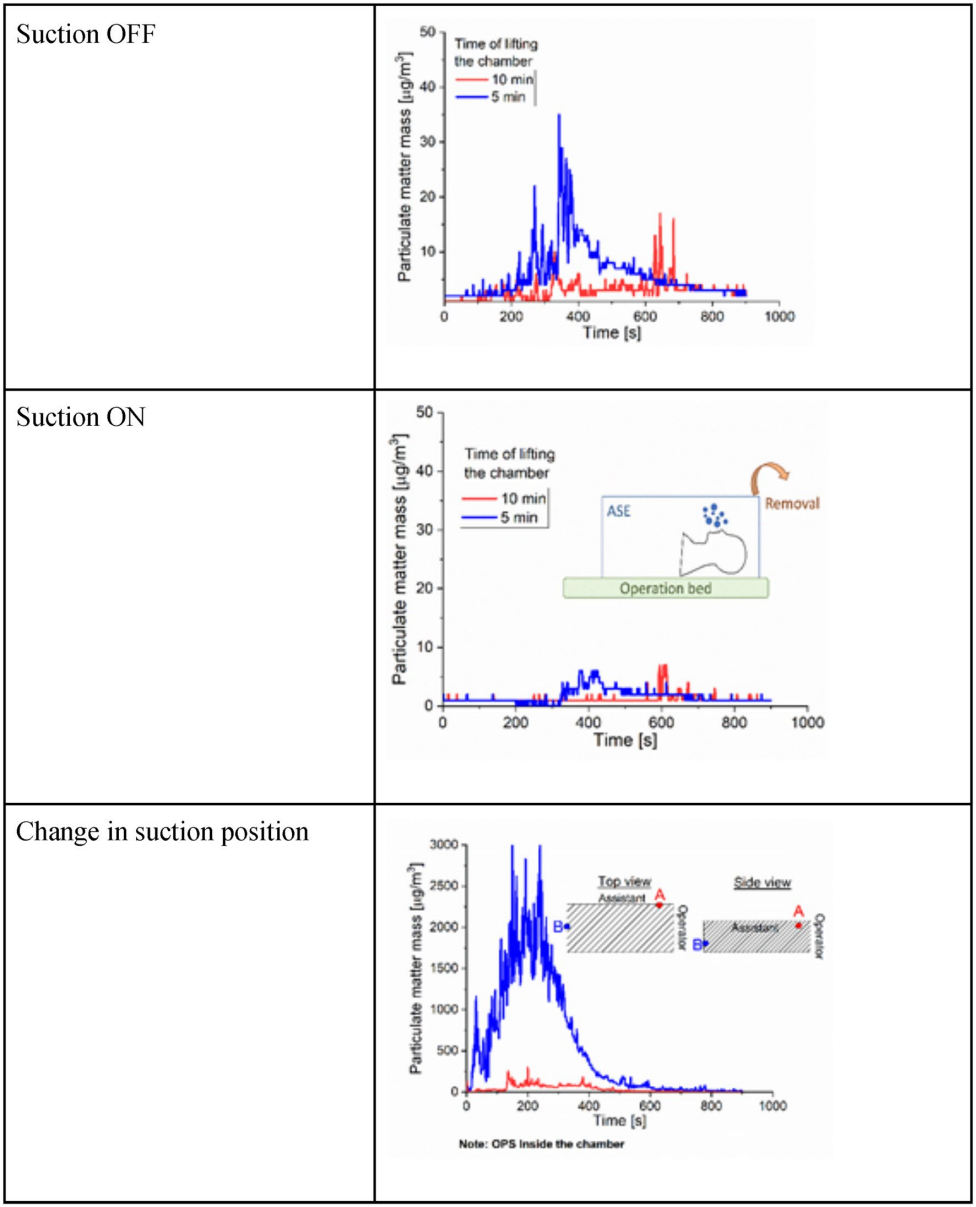
Average particulate matter recorded using an OPS. The event recorded involved the generation of aerosol for 5 min and then lifting of the Covid Surgery Box at the 5th and 10th minute. On the top row, no suctioning was involved, and in the second row, suction was running while the Box was positioned over the manikin. On the last row, the effect of changing the location of the suction port.

A manikin was modified to generate aerosols made of 0.9% normal saline solution from the oropharynx. To achieve this, one of the manikin’s lungs was connected to a nebulizer that produced droplets of saline solution at 10 L per minute. The manikin was positioned within the ASE in the typical set-up for an airway surgical procedure. Two types of devices were used to measure and quantify the aerosols: an Optical Particle Counter (OPC) and a low-cost sensor. The OPC model selected was the Dusttrak DRX aerosol monitor 8533 (TSI, Shoreview, Minnesota) which operates on the principle of light-scattering as particles pas between a light source and detector. It is commonly used as a reference method in measuring particle matter with a diameter between 0.3 and 10 µm^11^. This device could segregate the particulate matter into different size bins: diameters less than 10 µm (PM_10_), less than 4 µm (PM_4_), less than 2.5 µm (PM_2.5_), and less than 1 µm (PM_1_). The low-cost sensor model selected was uHoo (Milford, Ohio), which have been validated and used in previous studies^11^. These low-cost sensors are capable of measuring particulate matter with a diameter lower than 2.5 µm (PM_2.5_) using the same light-scattering method as the OPC. The detectable range and resolution are, respectively, 0 to 200 µg/m^3^ and 0.1 µg/m^3^. Low-cost sensors can be placed in an operating room and record in real-time, while OPC cannot. The droplet size distribution can be extracted by the baseline (no operation no box and aerosol on). Outliers from low-cost sensors were removed (the outliers usually is a reading of zero for a specific second). These experiments were conducted in an operating room with laminar airflow. The setup used to measure the airborne particulates around the ASE is demonstrated in Fig. 3. Three sensors were used; two units positioned outside the enclosure and one inside. The outside units were placed at the surgeon’s position (headend of enclosure) and at the assistant’s position (one side of the enclosure). Both of the sensors placed outside the ASE were at a height of 180 cm. The height was chosen to be above the head of an operator. A sensor lower (for example at the level of the mouth or nose) would have impeded the activities of the operators performing procedures so was not felt to be appropriate. The sensor inside the chamber was placed at the bottom side of the manikin, far from the assistant. The inlet of the OPS was located at the same position of the low-cost sensor close to the operator. A total of 17 unique testing conditions were studied and can be gathered in three main groups: baseline, using suction, and performing typical surgical events. Baseline conditions were when no events are occurring and 10 L min^−1^ of aerosolized particles were being generated. In the second group, the effect of the suction was tested while lifting the ASE. Therefore, the first five minutes showed the particulate being generated at baseline and the ASE was removed once at the 5th minute and once at the 10th minute. The same test was repeated with and without the suction running. Without removing the ASE, the position of the suction port was changed from a position lateral to one frontal with respect to the operator (Fig. 4). Preoxygenation, bag-masking, and positioning of an N95 respirator mask on the manikin were events used to test the shielding efficiency of the enclosure. The tests included the first 5 min with aerosol and events co-existing, and the remaining 10 min with no activity. Conditions with suction activated had this turned on for the whole length of the test. Every experiment lasted 15 min and was repeated 3 times.

### Steps for safe implementation into clinical care delivery

A step-wise and rigorous implementation process was developed and undertaken before the ASE was deployed for use in patient care. This arms-length independent group had expert representation and input from Surgery, Anesthesia, Medical Ethics, Patient Experience, Administration, Medical Leadership, Medical Device Reprocessing, and Infection Control. The first stage involved was simulation of candidate surgical procedures in a simulation lab using manikin ‘patients’ and operating room equipment, carefully identifying and documenting all workflow changes and potential risks and developing mitigation strategies. The next stage involved scenario-based simulation of surgical procedures in an Operating Room using a high-fidelity manikin, with a full medical team participation (anesthesiologists, surgeons, and nursing staff). Multiple scenarios were created and practiced. During the scenarios, pre-determined adverse events suddenly ‘occurred’ at the prompt of an arms-length expert, to test mitigation strategies that were developed during the lab-based simulations. For example, sudden respiratory failure requiring emergency intubation, and a sudden cardiac arrest requiring chest compressions. Through the performance of the lab and operating room-based simulations, written step-by-step protocols for safe implementation ASE were developed, including safety checklists and training materials.

### Simulation training

The assembled team consisted of two otolaryngologists, who had practiced extensively with the box in the lab environment; three surgical nurses with experience of airway procedures; and two anesthesiologists. Three simulated procedure scenarios were developed to be performed on appropriately-sized high-fidelity manikin: (1) suspension endo-laryngo-bronchoscopy for microdebridement of laryngeal papilloma in a 5-year-old; (2) diagnostic rigid bronchoscopy for stridor in a 16-week-old; and (3) rigid bronchoscopy for removal of airway foreign body in a 20-month-old. An arms-length otolaryngologist was engaged to be present throughout and provide oversight of the scenarios, and was tasked with developing spontaneous simulated adverse events for the team to manage during the procedures.

### Statement of institutional review board approval

The interventions in this study were approved by the BC Children’s hospital Innovative Procedure Safety Committee for deployment in patient care. All procedures contributing to this work comply with the ethical standards of the relevant national and institutional guidelines on human experimentation BC Children’s Hospital, University of British Columbia, Vancouver, Canada and with the Helsinki Declaration of 1975, as revised in 2008. Written informed consent was obtained from a parent and/or legal guardian of all study participants. What is notable is that the Airway Enclosure, together with other innovations designed specifically to protect operating room personnel, should be considered differently from other surgical innovations that typically aim to improve clinical outcomes for patients. Specifically, in contrast to surgical innovations where any risk to the patient can be weighed against benefits to the patient, somewhat paradoxically, the primary aim of the ASE is to protect hospital personnel with no obvious secondary benefit for the patient. Therefore, it is essential that the safety of the patient is kept at the forefront and risk to the patient is mitigated in utilization of the device.

## Results

### Early clinical experience

After successful simulations, the ASE was certified by the Hospital Innovative Procedure Safety Committee for deployment in patient care. Use of the ASE in clinical care included a full and detailed pre-operative discussion with the patient and family. Herein is a description of the implementation of ASE for its first four patients consisting three female and one male child. The patients were all tested as negative to SARS-CoV-2 within 24 h preceding the procedure The ages of the patients were 3 months (diagnostic rigid bronchoscopy and flexible transnasal endoscopy), 8 months (diagnostic rigid bronchoscopy) 5 years (suspension endo-laryngo-bronchscopy for removal of laryngeal papilloma), and 12 years (suspension endo-laryngo-bronchscopy for removal of laryngeal papilloma). Intravenous anaesthesia was commenced before the enclosure was positioned over the patient. The enclosure was placed over the patient prior to airway manipulation (e.g. laryngoscopy or administration or airway topical local anaesthesia). There were no adverse events encountered in any of the procedures and all were completed successfully. No deviations from the planned protocols were required. Procedures were timed and found to be within the typical range for each procedure (compared to the case lengths for the preceding 5 procedures for each procedure type), excluding the extended pre-operative huddle run-through, the 5 min ASE aerosol suction ‘washout’ period before removal at the end of surgery, and the time taken for allowing the patient to arouse from sedation within the operating room. Formal statistical comparison was not feasible due to small number or procedures performed. The specific time taken to position and remove the ASE were recorded for all the procedures and were as follows: placing ASE in position over the patient took an average of 24 s (range: 14 to 32 s); removing took an average of 10 s (range: 8 s to 11 s). Feedback from the operators was that there was a mild learning curve that was easily overcome with the simulations. It was not felt by any of the participants that this impeded their ability to safely perform the procedure and the added sense of exposure safety for all involved health care staff was of substantial benefit in their confidence to perform the procedures.

### Aerosolization testing results

**Fig. 5:**
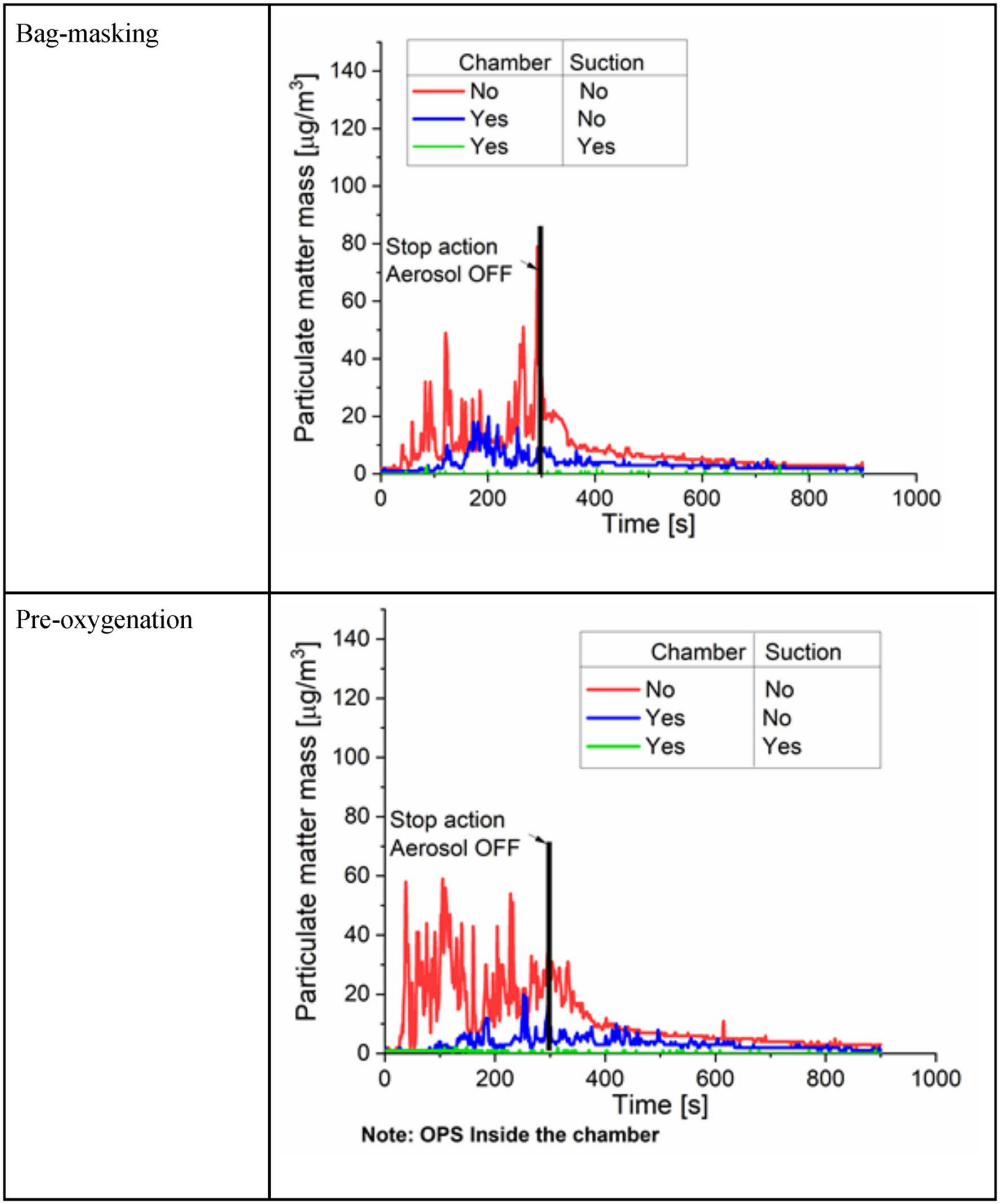
The effect of the use of the Covid Surgery Box and pre-oxygenation and bag masking the patient. Average Particulate Matter (PM) recorded with the OPS are shown.

Baseline measurements were taken for aerosol particulate matter (PM) in the operating room both with and without aerosol generation (‘no event’) from the manikin. At the surgeon location, the average PMs without aerosol generation were 1 µg/m^3^, but rose to a maximum of 115 µg/m^3^ with the aerosol generation, as shown in Table 1. The second group of experiments explored the effect of the ASE on aerosol in the operating room, including with removal of the ASE, which would be expected to occur once a surgical procedure is completed. After generating aerosol for 5 min, the ASE was lifted after 300 s and 600 s (i.e. immediately on shutting down aerosol generation or after waiting 5 min after shutting down the aerosol generation). Figure 4 illustrates the particulate matter recorded in these conditions; on the top row, no suction from the ASE was employed, and in the bottom row, suction was active while the ASE was in position over the manikin. The sensors demonstrated that any size of particulate matter had a sudden increase after the ASE was lifted, both at 300 and 600 s, when no suction was active (Table 1 and Fig. 4). As expected, the rise at 600 s was lower than the rise at 300 s (10 µg/m^3^ vs 30 µg/m^3^, respectively). The effect of the suction is clearly shown in the middle row of Fig. 4. The OPS recorded spikes below 10 µg/m^3^ of average PM. Regardless of the lifting time, the use of suction greatly reduced the exposure of the surgeon and assistant to any aerosol particulate matter escaping the ASE. In addition to evaluating the particulate matter escaping the ASE while simply generating aerosol, we tested the aerosol produced by events that may be seen during airway surgical procedures: patient pre-oxygenation and bag masking (Table 1 and Fig. 5). The events were conducted for the first 300 s of the trials. During these events, the aerosol was turned on and then off after 300 s.

**Table 1:** Summary of the average and the maximum values determined for each test and location.

Test type	Test #		Operator					Assistant	Inside
			PM_1_	PM_2.5_	PM_2.5_ LC	PM_4_	PM_10_	PM_2.5_ LC	PM_2.5_ LC
Baseline	No events	Ave	0	0	5	0	1	7	7
		Min	0	0	3	0	0	4	4
		Max	3	3	6	4	20	8	10
	Aerosol ON	Ave	12	14	5	15	16	6	7
		Min	0	0	3	0	0	3	4
		Max	113	126	11	126	127	8	21
Effect of the suction	Lift Chamber at 10 min No suction	Ave	3	3	2	3	7	8	6
		Min	0	1	0	1	1	6	3
		Max	17	17	11	27	83	15	9
	Lift Chamber at 5 min No suction	Ave	6	6	13	6	8	8	6
		Min	1	2	0	2	2	6	3
		Max	34	35	36	35	41	15	12
	Lift Chamber at 10 min Suction	Ave	1	1	6	1	3	6	129
		Min	0	1	3	1	1	4	2
		Max	7	7	7	7	28	8	200
	Lift Chamber at 5 min Suction	Ave	2	2	7	2	3	8	105
		Min	0	0	3	0	0	6	5
		Max	6	6	12	7	31	9	200
	Position A Suction	Ave	443	550	7	550	550	6	134
		Min	3	4	3	4	4	4	15
		Max	2160	3120	8	3130	3130	8	200
	Position B Suction	Ave	36	37	7	36	38	7	99
		Min	0	5	3	1	1	4	21
		Max	293	300	13	300	300	18	200
Event of Preoxygenation	No chamber No suction	Ave	11	12	11	12	13	4	74
		Min	2	5	7	1	1	0	8
		Max	55	59	23	59	59	8	200
	Chamber No suction	Ave	3	3	7	4	5	7	137
		Min	2	1	3	1	1	3	10
		Max	19	20	12	20	35	9	200
	Chamber Suction	Ave	1	1	6	1	1	6	150
		Min	0	0	3	0	0	4	8
		Max	2	2	8	4	26	8	200
Event of bag masking	No chamber No suction	Ave	9	9	7	9	10	10	51
		Min	2	2	3	2	2	5	5
		Max	78	79	12	79	79	23	200
	Chamber No suction	Ave	3	4	6	4	5	6	120
		Min	1	1	3	1	1	4	33
		Max	25	20	9	21	30	8	200
	Chamber Suction	Ave	1	1	6	1	1	6	154
		Min	0	0	3	0	0	4	77
		Max	3	3	7	3	41	7	200
Event of wearing an N95 respirator on the manikin	No chamber No suction	Ave	7	7	7	7	8	8	63
		Min	0	1	3	1	1	4	4
		Max	138	142	16	143	146	10	200
	Chamber No suction	Ave	4	5	7	4	5	7	50
		Min	1	1	3	1	1	3	4
		Max	15	15	8	15	32	11	138
	Chamber Suction	Ave	1	1	6	1	1	7	117
		Min	0	0	3	0	0	4	26
		Max	3	3	7	3	29	8	200

Units are in µg/m^3^. The term “LC” indicates low-cost sensor.

Clinically significant particle reduction thresholds are generally defined as a minimum 80% decrease in airborne particle concentration, as this level has been shown to substantially reduce the risk of contamination in surgical environments. Our study demonstrated that the ASE achieved an 87–94% reduction in aerosolized particles within the enclosure compared to ambient room air levels. These reductions were sustained across multiple procedural simulations, reinforcing the efficacy of the ASE in mitigating airborne risks.

## Discussion

We demonstrate a non-commercial, open-source (www.covidbox.ca) device that can be added to the arsenal of techniques described to reduce the risk of respiratory viral exposure in the operating room. Since the development of this box a number of other boxes, drapes and combined devices have been described and are summarized in reviews^6,12^. The limitations of these studies are that most are related to intubation and most have no simulated or real-world validation of particulate protection. We provided a comprehensive evaluation of the particulate protection and real-world application of this box and describe its development. The surgical and anesthetic teams all provided feedback during the study that the learning curve was minimal for performing the procedures with the assistance of the enclosure. All staff felt comfortable after one simulation (although multiple simulations were run as per the safety protocol). We hope that this will be of use to groups trying to develop similar technology in the future. This non-commercial and open-source design can be added to the arsenal of techniques described and available to otolaryngologists worldwide. It should be noted that in very poor infrastructure settings these enclosures cannot work as they need a pump. The use of this device, and/or future modified versions, will improve safety during high-risk, aerosol-generating procedures in any future pandemics. Future iterations of the ASE could incorporate advanced filtration systems, such as HEPA or UV-C light sterilization, to enhance containment efficiency further. Additionally, lightweight and foldable materials could be explored to improve portability while maintaining structural integrity. Integration with real-time aerosol monitoring could also provide immediate feedback on containment performance, facilitating real-world optimization.

Commercially available aerosol containment solutions, such as rigid intubation boxes and negative-pressure tents, have been widely implemented in clinical settings. These solutions provide varying degrees of protection but also present limitations. Rigid intubation boxes, commonly constructed from acrylic or polycarbonate, offer a physical barrier to airborne particles but suffer from limited access points, poor ergonomics, and difficulty in thorough decontamination. Negative-pressure tents effectively contain aerosolized particles by utilizing continuous airflow systems; however, their bulkiness, cost, and requirement for specialized infrastructure restrict widespread use. Compared to these solutions, the ASE provides a balance between accessibility, containment efficiency, and ease of sterilization while maintaining a negative-pressure environment tailored for airway procedures.

## Limitations

The use of barrier-enclosure devices adds to the complexity of airway procedures with potential adverse consequences, especially during airway emergencies. Concerns include limitations on the ability to perform airway interventions and the aid that can be delivered by an assistant, patient injuries, compromise of PPE integrity, lack of evidence for added protection of healthcare providers (including secondary aerosolisation upon barrier removal), and lack of cleaning standards. Enclosure barriers for airway management are no substitute for adequate PPE, and their use should be avoided until adequate validation studies can be reported.

The Airway Surgery Enclosure was designed for pediatric patients up to the age of approximately 14 years. The age of the patient is limited by the width of the patient’s shoulders and the use of arm holders connected to the operating room table. This limitation is because with its current design creating a seal against the mattress of the operating room table, the ASE cannot be wider than the operating room table itself. An adaption in the design and size of the enclosure would be required for successful use in most s, particularly shoulder girth and torso length. The exploratory design and safety validation work required for this was beyond the scope of this project. In the scenario of an airway foreign body removal, the lengths of the instruments must be considered. The trajectory of inserting the instruments like optical forceps must be reflected in the design in order to avoid the instrument colliding against the surgeon-facing wall of the CSE. Therefore, the distance from the patient’s head to the surgeon-facing wall must be sufficient and should be measured before starting the procedure. The need for potentially unanticipated airway instruments, such as forceps or balloons must be discussed at the huddle and may need to be introduced intraoperatively at the foot-end of the box by briefly breaking the seal. For the tested procedures no significant restrictions were identified in mobility. The use of a microscope with the ASE is not possible due to impeded positioning and optics, and only endoscopes can be used for airway surgery. Of note the operating video display unit had to be place at the appropriate height and angle so as it could be seen directly without having to look through the enclosure, I.e. in direct line of sight of the surgeon, which was slightly elevated from it typical position. The ergonomics of this screen position were found to be very acceptable for the surgical team.

For most pediatric airway procedures, patients in our centre are anesthetized using total intravenous anesthesia and spontaneously breathing. Supplemental oxygen could be provided through the ASE through a side port to fill the enclosure, which is an advantage over conventional anesthesia, where supplemental oxygen is usually only provided via a nasal in-situ tube, or through the laryngoscope and bronchoscope side ports. It is notable though that the use of laser or monopolar electro-cautery must be avoided in the above scenario in the ASE, due to the risk of an airway fire in an oxygen-rich environment. We also did not test scenarios in the ASE with a simulated cough and measurement of aerosols generated. Airway procedures can induce coughing, which may direct aerosols to specific locations in the ASE and influence the results. In our experience we find with total intravenous anesthesia and topical local anesthesia to the airway, patient cough during these procedures is an extremely rare event. Nevertheless, further validation of this scenario would be beneficial in the future.

Clear acrylic panels were selected due to their availability, low material cost, and ease of assembly compared to alternative materials. For future iterations, it is recommended to use polycarbonate or other similar materials with higher impact strength.

## Conclusions

We describe the safe implementation process and evidence of efficacy in reducing circulating aerosol from AGMPs using our box design. This has real world application, particularly in low resource settings and is available free as open source for further development and use by clinicians and researchers.

## Data Availability

All datasets used and/or analyzed during the current study can be obtained from the corresponding author upon reasonable request. We plan to publish the plans of the enclosure and associated designs/validation via a hyperlink prior to publication.

## References

[1] Zou, L. et al. SARS-CoV-2 viral load in upper respiratory specimens of infected patients. *N. Engl. J. Med.* **382**, 1177–1179. 10.1056/NEJMc2001737 (2020). 32074444 10.1056/NEJMc2001737 PMC7121626

[2] Kowalski, L. P. et al. COVID-19 pandemic: Effects and evidence-based recommendations for otolaryngology and head and neck surgery practice. *Head Neck* **42**, 1259–1267. 10.1002/hed.26164 (2020). 32270581 10.1002/hed.26164 PMC7262203

[3] Krajewska, J., Krajewski, W., Zub, K. & Zatonski, T. COVID-19 in otolaryngologist practice: a review of current knowledge. *Eur. Arch. Otorhinolaryngol.* **277**, 1885–1897. 10.1007/s00405-020-05968-y (2020). 32306118 10.1007/s00405-020-05968-y PMC7166003

[4] Prakash, L., Dhar, S. A. & Mushtaq, M. COVID-19 in the operating room: a review of evolving safety protocols. *Patient Saf. Surg.* **14**, 30. 10.1186/s13037-020-00254-6 (2020). 32695225 10.1186/s13037-020-00254-6 PMC7370871

[5] Canelli, R., Connor, C. W., Gonzalez, M., Nozari, A. & Ortega, R. Barrier enclosure during endotracheal intubation. *N. Engl. J. Med.* **382**, 1957–1958. 10.1056/NEJMc2007589 (2020). 32243118 10.1056/NEJMc2007589 PMC7151333

[6] Sorbello, M., Rosenblatt, W., Hofmeyr, R., Greif, R. & Urdaneta, F. Aerosol boxes and barrier enclosures for airway management in COVID-19 patients: a scoping review and narrative synthesis. *Br. J. Anaesth.* **125**, 880–894. 10.1016/j.bja.2020.08.038 (2020). 32977955 10.1016/j.bja.2020.08.038 PMC7470712

[7] Girgis, A. M. et al. Novel coronavirus disease 2019 (COVID-19) aerosolization box: Design modifications for patient safety. *J. Cardiothorac. Vasc. Anesth.* **34**, 2274–2276. 10.1053/j.jvca.2020.05.001 (2020). 32446708 10.1053/j.jvca.2020.05.001 PMC7201230

[8] Foster, P. et al. Novel approach to reduce transmission of COVID-19 during tracheostomy. *J. Am. Coll. Surg.* **230**, 1102–1104. 10.1016/j.jamcollsurg.2020.04.014 (2020). 32283268 10.1016/j.jamcollsurg.2020.04.014 PMC7146662

[9] Francom, C. R. et al. Pediatric laryngoscopy and bronchoscopy during the COVID-19 pandemic: A four-center collaborative protocol to improve safety with perioperative management strategies and creation of a surgical tent with disposable drapes. *Int. J. Pediatr. Otorhinolaryngol.* **134**, 110059. 10.1016/j.ijporl.2020.110059 (2020). 32339971 10.1016/j.ijporl.2020.110059 PMC7172675

[10] McDowell, M. A., Fryar, C. D. & Ogden, C. L. Anthropometric reference data for children and adults: United States, 1988–1994. *Vital Health Stat.* **11**, 1–68 (2009). 19642512

[11] Baldelli, A. Evaluation of a low-cost multi-channel monitor for indoor air quality through a novel, low-cost, and reproducible platform. *Meas. Sens.* **17**, 100059. 10.1016/j.measen.2021.100059 (2021).

[12] Ahmad, M. Protective boxes to prevent airborne transmission of SARS-COV-2: Hospital-based experiences and a narrative literature review. *Open Access Emerg. Med.* **13**, 355–362. 10.2147/OAEM.S314559 (2021). 34349570 10.2147/OAEM.S314559 PMC8326778

