# Correction: Development and validation of the airway surgery enclosure for high-risk aerosol-generating airway procedures: a bench and clinical study

**DOI:** 10.1038/s41598-025-12694-0

**Published:** 2025-07-25

**Authors:** Neil K. Chadha, Jason Powell, Katharina Leitmeyer, Mark Felton, Alberto Baldelli, Michael Rooney, Fraser G. L. Parlane, Robert Purdy

**Affiliations:** 1 Division of Pediatric Otolaryngology-Head and Neck Surgery, BC Children’s Hospital, University of British Columbia, 4480 Oak Street, Vancouver, BC Canada; 2 Translational and Clinical Research Institute, Faculty of Medical Sciences, Newcastle University, Newcastle Upon Tyne, UK; 3 Division of Pediatric Otolaryngology-Head & Neck Surgery, University Children’s Hospital of Basel, Basel, Switzerland; 4 Faculty of Food and Land Systems, University of British Columbia, Vancouver, Canada; 5 Department of Pediatric Anesthesia, BC Children’s Hospital, University of British Columbia, Vancouver, Canada; 6 Department of Chemistry, The University of British Columbia, Vancouver, BC Canada; 7 Stewart Blusson Quantum Matter Institute, The University of British Columbia, Vancouver, BC Canada

Correction to: *Scientific Reports* 10.1038/s41598-025-03705-1, published online 02 July 2025

The original version of this Article contained an error in the name of the author Katharina Leitmeyer, which was incorrectly given as Kathatina Leitmeyer.

The original Article has been corrected.

